# Variable stiffness locomotion with guaranteed stability for quadruped robots traversing uneven terrains

**DOI:** 10.3389/frobt.2022.874290

**Published:** 2022-08-29

**Authors:** Xinyuan Zhao, Yuqiang Wu, Yangwei You, Arturo Laurenzi, Nikos Tsagarakis

**Affiliations:** ^1^ Humanoid and Human Centered Mechatronics Research Line, Istituto Italiano di Tecnologia, Genova, Italy; ^2^ Corporate Research Center, Mechatronics Research Institute, Midea Group Co., Ltd., Foshan, China; ^3^ Xiaomi, Beijing, China

**Keywords:** quadruped robots, locomotion control, variable stiffness control, touchdown adaptation, contact force optimization

## Abstract

Quadruped robots are widely applied in real-world environments where they have to face the challenges of walking on unknown rough terrains. This paper presents a control pipeline that generates robust and compliant legged locomotion for torque-controlled quadruped robots on uneven terrains. The Cartesian motion planner is designed to be reactive to unexpected early and late contacts using the estimated contact forces. Moreover, we present a novel scheme of optimal stiffness modulation that aims to coordinate desired compliance and tracking performance. It optimizes joint stiffness and contact forces coordinately in a quadratic programming (QP) formulation, where the constraints of non-slipping contacts and torque limits are imposed as well. In addition, the issue of stability under variable stiffness control is solved by imposing a tank-based passivity constraint explicitly. We finally validate the proposed control pipeline on our quadruped robot CENTAURO in experiments on uneven terrains and, through comparative tests, demonstrate the improvements of the variable stiffness locomotion.

## 1 Introduction

Quadruped robots have found extensive applications in real-world scenarios such as field exploration, industrial inspection, logistics and delivery, and disaster rescue (Bledt et al., 2018a; Bellicoso et al., 2018; Klamt et al., 2018; Lee et al., 2020) due to their better mobility in unstructured environments. They are often required to traverse rough terrains robustly in all of those applications. Even when cameras or lidars are available, the robots are still expected to adapt to some uncertainties in terrain geometries introduced by limited accuracy of the perception.

In case of unexpected interactions, impedance control (Hogan, 1985) is able to generate compliant behaviors by directly specifying the stiffness and damping that the system manifests to external disturbances. Instead of using constant joint stiffness, however, humans are found to keep modulating joint stiffness when performing daily tasks, enabling us to interact smoothly and stably with environments (Lee and Hogan, 2014; Wu et al., 2020a). It has also been demonstrated that robots may benefit from variable stiffness control in performing practical tasks as well. Whereas, the design of appropriate stiffness profiles that fulfill feasible requirements and optimality is still an open research topic.

Different approaches have been studied in the literature to implement variable stiffness control. An exponential-function-based modulation of joint stiffness is implemented in (Zollo et al., 2003), where the robot is required to be more compliant when the tracking errors of joint positions increase so that it achieves safe collision with the environment. Reinforcement learning has been a common practice in the field of robotics to obtain complex behaviors and it is used in (Heijmink et al., 2017) to generate optimal stiffness trajectories for quadruped robots. The policy is trained offline in simulations, trading off tracking performance, energy consumption and fall avoidance, and then validated on the robot HyQ. Another type of learning algorithm, namely imitation learning, is employed in (Wu et al., 2020b) to copy the properties of variable stiffness of human manipulations to a robotic arm. The proposed method is shown to provide better tracking and interaction behaviors in comparative experiments. In contrast, optimization-based approaches, e.g., (Albu-Schäffer et al., 2004; Petit and Albu-Schäffer, 2011), do not require large data sets or human demonstrations. In (Petit and Albu-Schäffer, 2011), the authors propose to utilize both passive and active joint stiffness to achieve the desired Cartesian stiffness as much as possible. Two separate optimizations are therefore established to compute first the passive stiffness, which are decoupled among the joints, and then the active stiffness.

From the perspective of energy, modulating the stiffness of a robot may inject too much energy into it, which will lead the robot to instability. The works mentioned above, however, do not take into account stability issues in variable stiffness control. To this end, several approaches have been studied in the literature. An energy tank-based method is introduced in (Ferraguti et al., 2013) to analyze the passivity of manipulators under variable stiffness control. The energy dissipated from the system, which to some extent expresses the passivity margin, is stored in the tank and then applied to avoid injecting too much energy into the system by restricting the variation rate of the stiffness. Alternatively, the stability of variable stiffness control is proved by a Lyapunov-based method in (Kronander and Billard, 2016). It proposes to relate the variation rate of the stiffness to the value of damping and provide a systematic way to design the impedance parameters, which guarantees that the desired impedance can be achieved while complying with stability. (Angelini et al., 2019) proposes a variable stiffness controller for legged robots with guaranteed stability. By properly decoupling the dynamics in single-input and single-output (SISO) sub-systems, the authors manage to ensure the stability under variable stiffness control by restricting the variation rate of the stiffness.

In this paper, we present a control pipeline for torque-controlled quadruped robots. It features optimal modulation of joint stiffness with guaranteed stability and touchdown adaptation that allows overcoming uncertainties in terrain geometries using only proprioceptive information. To be specific, the motion planner relies on estimated contact forces to transit between gait phases, which endows the robot with adaptations to early or late contacts in unknown environments. Moreover, we propose a QP-based optimization over both contact forces and joint stiffness based on desired motions and desired Cartesian compliance. An energy tank-based approach is exploited as a passivity constraint to restrict the variation rate of the joint stiffness, thus ensuring stable behaviors of the robot. Other types of constraints such as unilaterality of contact forces and torque limits are also considered in the optimization to ensure practically feasible results. The contributions of this paper are summarized as follows.

**FIGURE 1:**
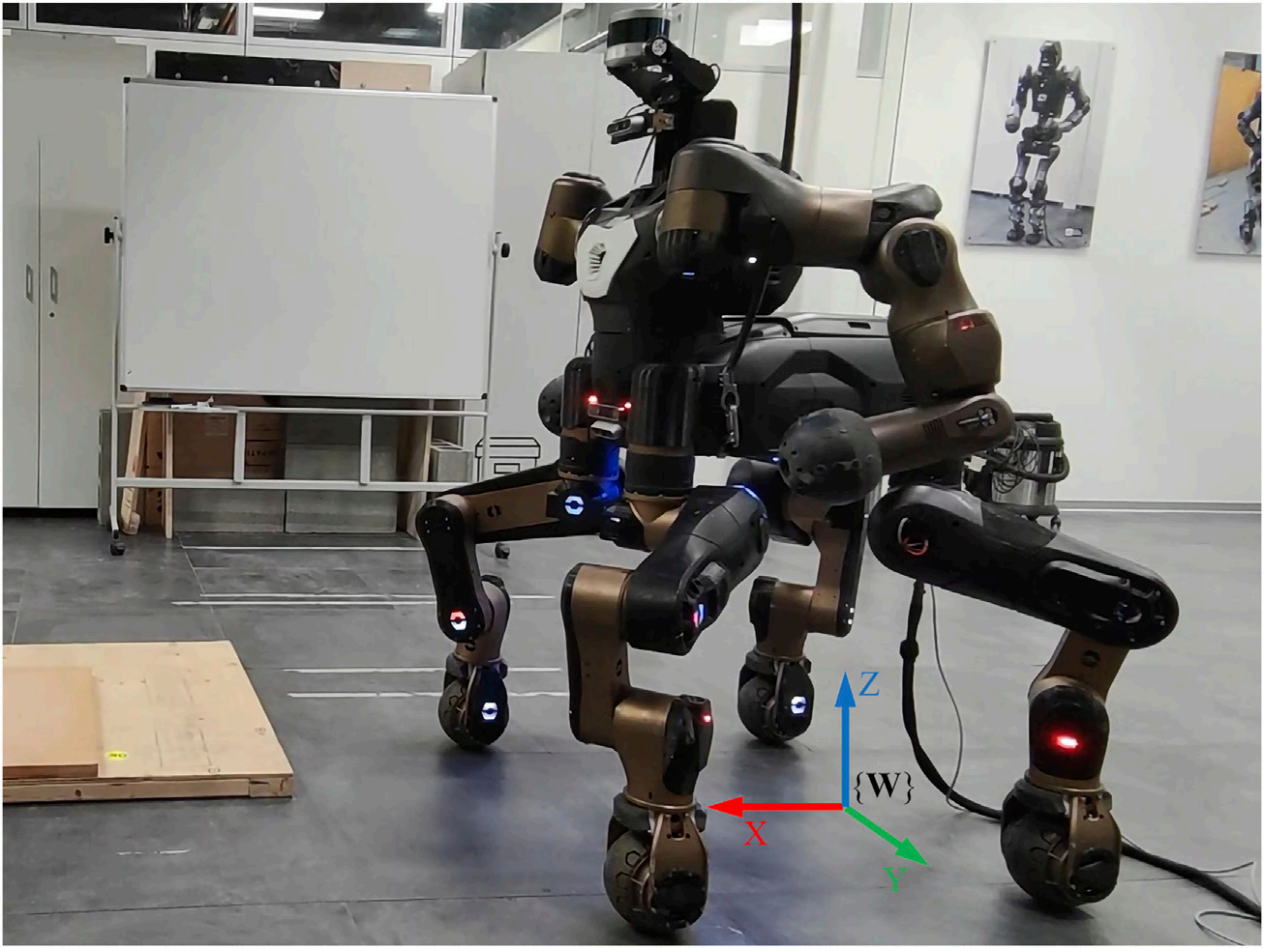
CENTAURO is a torque-controlled wheeled-legged quadruped robot designed in our lab (Humanoids and Human Centered Mechatronics, HHCM) at IIT. The world coordinate frame {**W**} is also displayed in the picture.

• A novel scheme for optimal stiffness modulation is proposed, where the desired stiffness gains and the desired contact forces are generated coordinately within a single optimization problem.
• The stability issue of stiffness modulation is resolved theoretically by introducing an energy tank-based constraint to the coordinated optimization. To the best of the authors’ knowledge, this is the first time that such a scheme with guaranteed stability is proposed and applied to quadruped robot locomotion.
• A control pipeline for torque-controlled quadruped robots, integrating the aforementioned scheme, is developed, enabling locomotion on uneven terrains with optimal modulation of joint stiffness. It is validated extensively on the quadruped robot CENTAURO (seen in Figure 1) through comparative tests.

The remainder of this paper is organized as follows. Section 2 covers approaches to generate position-based and torque-based legged locomotion for quadruped robots. Section 3 analyzes the influence of variable stiffness and then introduces our design of the coordinated optimization over contact forces and joint stiffness. Section 4 demonstrates experimental results that validate the proposed scheme. In Section 5, we discuss and compare this work with some related works and propose future directions as well.

## 2 Approaches of locomotion generation

**FIGURE 2:**
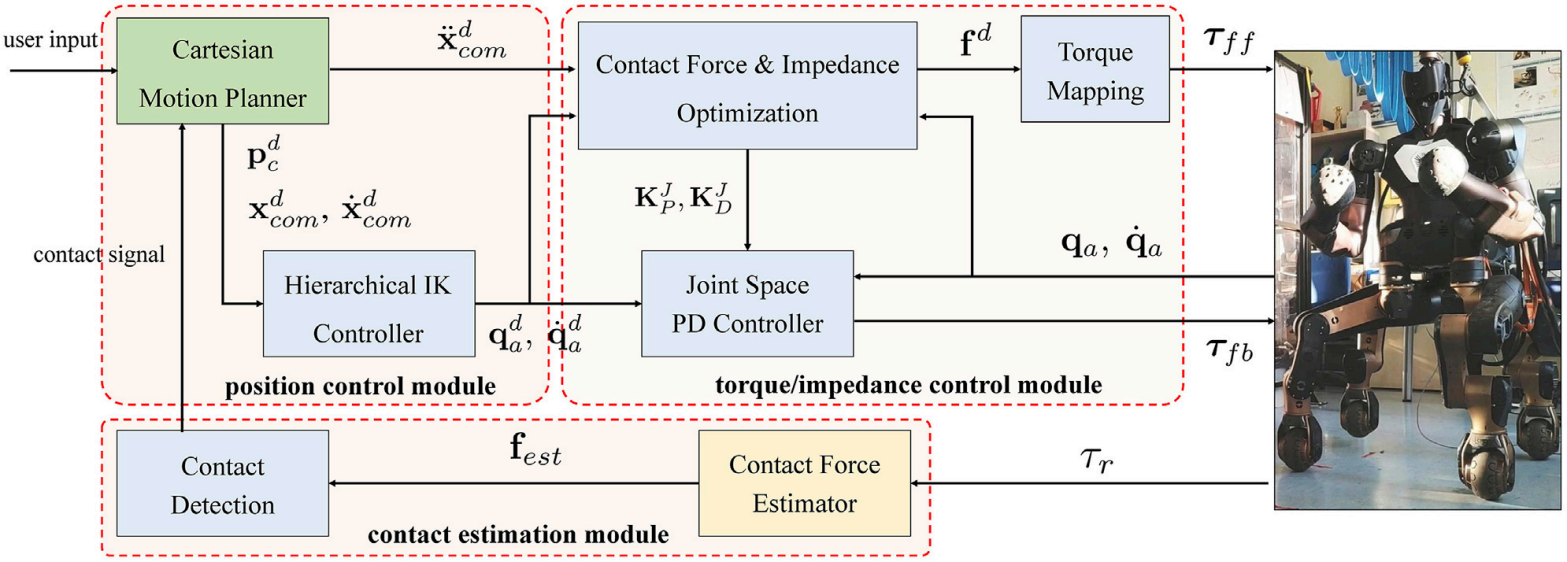
An overview diagram of the proposed control pipeline for legged locomotion. The dashed boxes represent the position control module, the torque/impedance control module and the contact estimation module, respectively. The color of blocks indicates execution frequency: the block with green shade runs at 10 Hz, the blocks with blue shade run at 200 Hz and the block with yellow shade runs at 400 Hz.

The proposed control pipeline for legged locomotion mainly consists of three modules: 1) a position control module that generates Cartesian reference trajectories and the corresponding joint motions; 2) a torque/impedance control module that computes feedforward and feedback torque commands; and 3) a contact estimation module that estimates ground contact forces and determines the actual contact states. An overview diagram of the proposed scheme is shown in Figure 2.

### 2.1 Cartesian motion planner

The generation of the reference motions for the center of mass (CoM) majorly follows our previous work (Zhao et al., 2021) and it will be recapitulated here. The robot dynamics is described by the linear inverted pendulum (LIP) model, where we focus on the motions of the CoM and the zero-moment-point (ZMP) but ignore the forces acting on the robot. Let **x**_*com*_ and **x**_*zmp*_ denote the positions of the CoM and the ZMP, respectively, in the world frame, and then the reference motions of the CoM are generated by optimizing the jerks ${\overset{...}{x}}_{\mathrm{com}}$ in an MPC fashion in the following formulation.

$$\min\overset{k_{0}+N}{\underset{k=k_{0}+1}{\sum }}\left(\frac{\alpha }{2}\|{\overset{...}{x}}_{\mathrm{com}}\left(t_{k}\right){\|}^{2}+\frac{\beta }{2}\|x_{\mathrm{zmp}}\left(t_{k}\right)-x_{\mathrm{zmp}}^{\mathrm{ref}}\left(t_{k}\right){\|}^{2}+\frac{\gamma }{2}\|x_{b}\left(t_{k}\right)-x_{b}^{\mathrm{ref}}\left(t_{k}\right){\|}^{2}\right)$$ (1)

$$\text{s.t.}\;x_{\mathrm{zmp}}\left(t_{k}\right)\in \text{ConvHull}\left\{p_{c,i}\left(t_{k}\right)\right\},\;i\in S\left(t_{k}\right)$$ (1a)

There are three objectives in the optimization (Eq. 1). The first objective is to minimize the CoM jerks. The second objective is to control the ZMP to track the reference $x_{\mathrm{zmp}}^{\mathrm{ref}}$ that is selected to be the center of the support polygon (SP). **x**_*b*_ in the last objective denotes the geometric center of the robot base and the reference $x_{b}^{\mathrm{ref}}$ is selected to be the geometric center of the convex hull formed by all the feet, regardless of their contact states. This objective is added to modulate the lower body away from leg singularities. **p**_*c*,*i*_ denotes the positions of the feet and $S\left(t_{k}\right)$ denotes a set of indices for the stance legs at this moment. Hence, $\text{ConvHull}\left\{p_{c,i}\left(t_{k}\right)\right\}$ represents the convex hull formed by the feet in contact, i.e., the SP. The constraint Eq. 1a is to place the ZMP always within the SP, which is the ZMP-based stability criteria for quasi-static locomotion.

Taking into account that CENTAURO weighs about 110 kg and is driven by actuators with high gear ratios, the robot adopts a quasi-static gait pattern in the locomotion tasks. The gait schedule of the swing leg is periodic, which follows the sequence left-front leg (LF) → right-back leg (RB) → right-front leg (RF) → left-back leg (LB). The feet in contact are required to keep stationary, while the reference motions of the swing foot are formulated by two segments of quintic polynomials, one for the lifting phase and the other for the landing phase.

#### 2.1.1 Touchdown adaptation

In this work, it is assumed that visual perception is not integrated to the locomotion controller. In order to adapt to uneven terrains that are unknown *a priori* in blind locomotion, contact-triggered touchdown adaptation is implemented.

**FIGURE 3:**
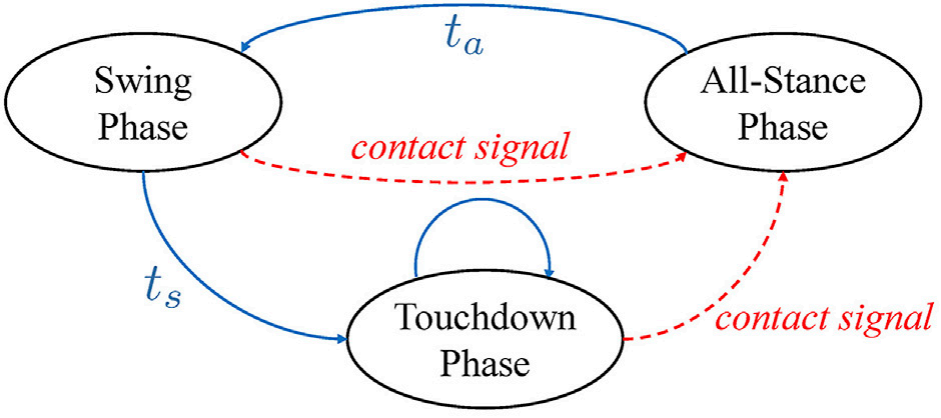
The finite state machine adopted by the Cartesian motion planner. The ellipses represent the three planning phases with the arrows indicating the possible transitions between them. *t*_*a*_ and *t*_*s*_ denote the periods of the All-Stance Phase and the Swing Phase, respectively. The dashed red arrows, however, indicate the time-independent transitions that can only be triggered by the actual contact state.

The motion planner adopts a finite state machine with time/event-driven transitions as illustrated in Figure 3. The blue arrows represent time-based transitions, where *t*_*a*_ and *t*_*s*_ denote the periods of the All-Stance Phase and the Swing Phase, respectively. The red dashed arrows represent transitions that can only be triggered by detected contact events. The motion of the swing foot is adjusted accordingly in the cases of early touchdown and late touchdown. When contact is detected in advance of the nominal moment, the swing foot is switched to contact state and the nominal reference motion is truncated immediately. On the other hand, if expected touchdown is not established within the duration *t*_*s*_, the swing foot will move downwards at a constant speed so as to search for contact. When contact is detected, in both of the two cases, the motion planner will then switch to the All-Stance Phase, update the internal model with the actual configurations and start a new planning loop. The adaptation to early and late touchdown implemented in this chapter will enable perception-less locomotion over moderately uneven grounds.

The contact event is detected by a threshold trigger of the estimated ground reaction forces, the calculation of which will be introduced later in Section 2.4. When the state remains in the Swing Phase and the current swing leg generates a reaction force larger than a specific threshold *f*_*up*_ on the *z*-axis, the contact state of that leg will switch from swing to contact and a contact signal will be sent simultaneously to trigger the state machine shown in Figure 3.

### 2.2 Whole-body IK controller

The Cartesian reference motions are then transformed into joint space by the whole-body controller which solves an inverse kinematics (IK) problem at 200 Hz. The IK problem is formulated as a hierarchical QP-based optimization inside *CartesI/O* (Laurenzi et al., 2019), a ROS-based framework for Cartesian control that facilitates the assignment of different tasks and constraints in a user-friendly manner.

To be specific, the whole-body IK controller used in our control pipeline solves a cascade of two constrained optimization problems (*i* = 1, 2) in the following formulation.

$${\dot{q}}_{i}^{*}=\underset{{\dot{q}}_{i}}{\mathrm{arg min}}\|J_{i}{\dot{q}}_{i}-{\dot{x}}_{i}{\|}^{2}+\lambda \|{\dot{q}}_{i}{\|}^{2}$$ (2)

$$\text{s.t.}\;{\underline{c}}_{i}\leq C_{in,i}{\dot{q}}_{i}\leq {\bar{c}}_{i}$$ (2a)

$$J_{1}{\dot{q}}_{1}^{*}=J_{1}{\dot{q}}_{2}\;\left(\text{only for }i=2\right)$$ (2b)

where the problem with *i* = 1 is assumed to be superior to the problem with *i* = 2. The first objective in Eq. 2 is to achieve the predefined task while the second term is to regularize the joint velocities. ${\dot{x}}_{i}\in R^{m_{i}}$ is the desired task velocities of the *i*-th problem and $J_{i}\in R^{m_{i}\times \left(n+6\right)}$ denotes the associated task Jacobian. ${\dot{q}}_{i}={\left[{\dot{q}}_{u,i}^{\text{T}}\;{\dot{q}}_{a,i}^{\text{T}}\right]}^{\text{T}}\in R^{n+6}$ denotes the velocities of the generalized state, including the 6 degree-of-freedoms (DoFs) of the floating base ${\dot{q}}_{u}\in R^{6}$ and the *n* DoFs of the actuated joints ${\dot{q}}_{a}\in R^{n}$ . The regularization factor *λ* is selected to be *λ* = 1 × 10^–4^ in our implementation. The inequality constraint Eq. 2a expresses the boundary limits of joint positions and joint velocities, and is imposed on the two optimization problems in order to produce mechanically feasible solutions. The equality constraint Eq. 2b expresses the requirement of prioritized tasks and is only imposed on the second problem. ${\dot{q}}_{i}^{*}$ denotes the optimal solution of the first optimization, and thus Eq. 2b implies that the secondary problem will be solved without affecting the primary problem.

Based on the robot model, the tasks and the constraints assigned by users, the specific form of the optimization Eq. 2 is constructed by *CartesI/O* where the matrices and the vectors involved are automatically generated, which greatly facilitates development and deployment of the controller. The optimization problems Eq. 2 are then solved by the *qpOASES* solver at each control loop. Readers are recommended to refer to the website^1^ for more about the algorithms, implementations and applications of *CartesI/O*, whereas the details will be skipped in this paper. The tasks and constraints used in our experiments are summarized in Table 1.

**TABLE 1:** Tasks and constraints assigned in the whole-body IK controller.

Task/Constraint Description	Priority
CoM tracking Cartesian references	*i* = 1
base orientation regulated to be constant	*i* = 1
legs tracking Cartesian references	*i* = 1
configuration of upper body regulated to be constant	*i* = 2
joint position constraint	-
joint velocity constraint	-

### 2.3 Contact force optimization

As shown in Figure 2, the robot is controlled by a combination of the feedforward torque $\tau_{ff}\in R^{n}$ and the feedback torque $\tau_{fb}\in R^{n}$ . Several approaches have been proposed in the literature to compute the torque references towards achieving the desired Cartesian motions. Some researchers propose to first optimize the contact forces through a QP-based problem and then map them into joint space to obtain torques commands (Ott et al., 2011; Gehring et al., 2013; Focchi et al., 2017), or through another inverse dynamics optimization (Stephens and Atkeson, 2010). It is also possible to bypass the computation of contact forces by directly mapping the desired motions into the space of joint torques through, e.g., orthogonal projections (Mistry et al., 2010; Winkler et al., 2017). Alternatively, the torque references can be computed by exploiting the full-body inverse dynamics (Sentis et al., 2010; Righetti et al., 2013). In this paper, we adopt a similar approach as in (Focchi et al., 2017; Mastalli et al., 2017) to compute ***τ***_*ff*_, which will be briefly summarized in the following part.

To start with, the *Centroidal* dynamics (Orin et al., 2013) is adopted to describe the relationship between the motion of the robot and the acting forces. The model assumes that the robot walks slowly and the legs stay close to their nominal configurations so that the model description is independent of specific joint positions, which is the case for CENTAURO in quasi-staic locomotion. We further assume that the angular velocity of the robot is approximated by the angular velocity of the base and the point-like contacts of CENTAURO do not generate ground reaction torques. Let ${\ddot{x}}_{\mathrm{com}}\in R^{3}$ , ${\dot{\omega }}_{b}\in R^{3}$ and $f_{i}\in R^{3}$ denote the acceleration of the CoM, the angular acceleration of the robot base and the ground reaction forces, respectively, and then the Centroidal dynamics is established as follows.

$$m\left({\ddot{x}}_{\mathrm{com}}-g_{c}\right)=\overset{n_{c}}{\underset{i=1}{\sum }}f_{i}$$ (3)

$$I_{G}{\dot{\omega }}_{b}+{\dot{I}}_{G}\omega_{b}=\overset{n_{c}}{\underset{i=1}{\sum }}\left(p_{\mathrm{com,i}}\times f_{i}\right)$$ (4)

where *n*_*c*_ is the number of feet in contact (in our setup of quasi-static locomotion, there is always *n*_*c*_ = 3 or *n*_*c*_ = 4). *m* is the mass of the robot. **g**_*c*_ = [0,0,−9.81]^T^ m/s^2^ is the gravity acceleration vector. $I_{G}\in R^{3\times 3}$ is the centroidal rotational inertia computed at the CoM. $p_{\mathrm{com,i}}\in R^{3}$ is the vector going from the CoM to the *i*-th foot. The equations above can be rewritten in matrix form as

$$\underset{A_{f}\in R^{6\times 3n_{c}}}{\underset{︸}{\left[\begin{array}{ccc} I_{3\times 3} & \ldots & I_{3\times 3}\\ {\left[p_{\mathrm{com,1}}\right]}_{\times } & \ldots & {\left[p_{com,n_{c}}\right]}_{\times } \end{array}\right]}}\underset{f\in R^{3n_{c}}}{\underset{︸}{\left[\begin{array}{c} f_{1}\\ \vdots \\ f_{n_{c}} \end{array}\right]}}=\underset{b_{f}\in R^{6}}{\underset{︸}{\left[\begin{array}{c} m\left({\ddot{x}}_{\mathrm{com}}-g_{c}\right)\\ I_{G}{\dot{\omega }}_{b}+{\dot{I}}_{G}\omega_{b} \end{array}\right]}}$$ (5)

where $I_{3\times 3}\in R^{3\times 3}$ denotes an identity matrix and ${\left[\cdot \right]}_{\times }\in R^{3\times 3}$ denotes the skew-symmetric matrix related to vector cross products, i.e., $v_{1}\times v_{2}={\left[v_{1}\right]}_{\times }v_{2},\;\forall v_{1},v_{2}\in R^{3}$ . Since the robot moves slowly in our experiments and the base orientation has been regulated to be constant by the whole-body IK controller (seen in Section 2.2), it is reasonable to assume that ${\dot{\omega }}_{b}=\omega_{b}=0$ in the following computation. This problem has six equations and at least nine free variables and thus always has infinite solutions. The redundancy can be exploited to satisfy the inequality constraints imposed by friction cones and the unilaterality requirements of contact forces.

The following optimization is solved at every control loop to compute the desired contact forces.

$$f^{d}=\underset{f}{\mathrm{arg min}}\alpha_{1}\|A_{f}f-b_{f}{\|}^{2}+\alpha_{2}\|f{\|}_{W}^{2}$$ (6)

$$\text{s.t.}\;d_{f,\min}\leq C_{f}f\leq d_{f,\max}$$ (6a)

where ‖ ⋅‖ stands for the 2-norm of a vector, and *α*_1_, *α*_2_ are two positive weight factors. The inequality Eq. 6a includes the constraints of friction cones and unilaterality of contact forces (Focchi et al., 2017). It is worth noting that the inequality Eq. 6a also constraints the desired contact forces generated by the swing leg to be 0 so that the dimensions of variables become constant, i.e., $f\in R^{12}$ and $A_{f}\in R^{6\times 12}$ in the optimization Eq. 6, which simplifies implementation in code and further extension as will be introduced in Section 3.2. The positive-definite weight matrix $W\in R^{12\times 12}$ is chosen as follows to minimize joint torques rather than contact forces

$$W=J_{c}S^{\text{T}}SJ_{c}^{\text{T}}$$ (7)

where $S=\left[0_{n\times 6}\;I_{n\times n}\right]$ is a matrix selecting the actuated *n* DoFs and $J_{c}\in R^{12\times \left(n+6\right)}$ is the contact Jacobian.

The feedforward torque ***τ***_*ff*_ is finally obtained by mapping the optimal contact forces **f** ^*d*^ into joint space through

$$\tau_{ff}=S\left(h-J_{c}^{\text{T}}f^{d}\right)$$ (8)

where $h=C\left(q,\dot{q}\right)\dot{q}+g\left(q\right)\in R^{\left(n+6\right)}$ represents the non-linear dynamics caused by centripetal, Coriolis and gravitational forces. Besides the feedforward torque, there is also a feedback controller running in parallel to compensate for the tracking errors caused by model mismatch or interactions. The feedback torque ***τ***_*fb*_ is calculated as follows

$$\tau_{fb}=K_{P}\left(q_{a}^{d}-q_{a}\right)+K_{D}\left({\dot{q}}_{a}^{d}-{\dot{q}}_{a}\right)$$ (9)

where the diagonal, positive-definite matrices $K_{P},K_{D}\in R^{n\times n}$ represent the stiffness and damping matrices in joint space, respectively.

### 2.4 Contact detection

CENTAURO is not equipped with foot force/torque sensors and, therefore, the ground reaction forces **f** ^*est*^ are estimated from the measurements of joint torques and the dynamics of the robot through

$$f_{i}^{\mathrm{est}}=J_{c,i}^{{\text{T}}^{+}}\left(M\ddot{q}+h-S^{\text{T}}\tau_{r}\right),\;i=1,\ldots ,4$$ (10)

where $J_{c,i}\in R^{3\times \left(n+6\right)}$ represents the Jacobian of the *i*-th leg and (⋅)^+^ denotes the pseudo-inverse operation. $M\in R^{\left(n+6\right)\times \left(n+6\right)}$ denotes the joint-space inertial matrix of the robot. $\tau_{r}\in R^{n}$ represents the measurements of torques. The matrix **S** and the vector **h** are the same as defined in Eq. 8.

Due to noisy measurements from the sensors and inaccurate parameters of the model, the contact forces estimated by Eq. 10 usually suffer from non-negligible noise. To mitigate such effects, a first-order low-pass filter with cutoff frequency of 20 Hz is adopted to smooth the estimation. Although the contact force estimation is implemented in a simple manner compared with, e.g. (Bledt et al., 2018b) or (Camurri et al., 2017), we have validated in experiments that it is able to provide good enough results for our robot in quasi-static locomotion.

## 3 Optimal stiffness modulation

The proposed scheme of optimal stiffness modulation will be introduced in detail in this section. First of all, it is worth clarifying that the stability of variable stiffness control is different from the locomotion stability for quadruped robots (the latter is ensured by the ZMP constraint as introduced in Section 2.1). It concerns whether the modulation of stiffness gains will inject too much potential energy into the robot and drives the system to divergence. This section starts by analyzing the influence of variable stiffness control from the perspective of energy, followed by our scheme of stiffness optimization with guaranteed stability.

### 3.1 Stability analysis of variable stiffness control

In this section, we will analyze the stability issues of variable stiffness control from the perspective of energy, following a similar approach as in (Ferraguti et al., 2013; Kronander and Billard, 2016), and provide a sufficient condition that theoretically ensures stability under variable stiffness control. The analysis is carried out by considering each limb of the robot separately, which can be modeled as an articulated chain with *l* joints as follows (Siciliano et al., 2009)

$$M_{l}\left(q_{l}\right){\ddot{q}}_{l}+C_{l}\left(q_{l},{\dot{q}}_{l}\right){\dot{q}}_{l}+g_{l}\left(q_{l}\right)=\tau_{l}+J_{c,l}^{\text{T}}f_{l}$$ (11)

where $q_{l}\in R^{l}$ denotes the joint positions. $M_{l}\in R^{l\times l}$ denotes the symmetric and positive-definite inertia matrix in joint space. $C_{l}\in R^{l\times l}$ denotes the Coriolis/centrifugal matrix that is selected to fulfill the property

$$v^{\text{T}}\left({\dot{M}}_{l}\left(q_{l}\right)-2C_{l}\left(q_{l},{\dot{q}}_{l}\right)\right)v=0,\;\forall v,q_{l},{\dot{q}}_{l}\in R^{l}$$ (12)

which implies that ${\dot{M}}_{l}-2C_{l}$ is a skew-symmetric matrix. $g_{l}\in R^{l}$ denotes the torques due to gravitational effects. $\tau_{l}\in R^{l}$ denotes the control torques and $f_{l}\in R^{3}$ denotes the external forces, where **J**_*c*,*l*_ represents the associated Jacobian. The dependence on variables will be dropped in the following part for convenience.

The goal of impedance control is to regulate the relationship of the motions of the robot and external interactions. Let ${\tilde{q}}_{l}=q_{l}-q_{l}^{d}$ denote the errors between the actual and the desired joint positions. Then the closed-loop dynamics under the control of the feedforward and feedback torques is derived as follows

$$M_{l}{\ddot{\tilde{q}}}_{l}+\left(K_{D,l}+C_{l}\right){\dot{\tilde{q}}}_{l}+K_{P,l}{\tilde{q}}_{l}=\underset{{\tilde{\tau }}_{e}}{\underset{︸}{J_{c,l}^{\text{T}}\left(f_{l}-f_{l}^{d}\right)}}$$ (13)

where $K_{P,l}\in R^{l\times l}$ and $K_{D,l}\in R^{l\times l}$ denote the corresponding submatrices of the joint stiffness matrix **K**_*P*_ and damping matrix **K**_*D*_, respectively. In this work the desired joint accelerations ${\ddot{q}}_{l}^{d}=0$ .

To check the energy variation under the impedance control law, a storage function is selected to be

$$V=\frac{1}{2}{\dot{\tilde{q}}}_{l}^{\text{T}}M_{l}{\dot{\tilde{q}}}_{l}+\frac{1}{2}{\tilde{q}}_{l}^{\text{T}}K_{P,l}{\tilde{q}}_{l}$$ (14)

and what follows after derivative w.r.t time and substituting Eq. 13 is

$$\dot{V}={\dot{\tilde{q}}}_{l}^{\text{T}}{\tilde{\tau }}_{e}+\left(\frac{1}{2}{\tilde{q}}_{l}^{\text{T}}{\dot{K}}_{P,l}{\tilde{q}}_{l}-{\dot{\tilde{q}}}_{l}^{\text{T}}K_{D,l}{\dot{\tilde{q}}}_{l}\right)$$ (15)

where the property $\dot{M}-2C$ being a skew-symmetric matrix has be applied. If the term inside the parentheses in Eq. 15 is not positive, there will be

$$V\left(t\right)-V\left(0\right)\leq \int_{0}^{t}{\dot{\tilde{q}}}_{l}^{\text{T}}{\tilde{\tau }}_{e}\text{d}t$$ (16)

implying that the closed-loop system Eq. 13 is passive w.r.t. The input-output pair $\left({\tilde{\tau }}_{e},{\dot{\tilde{q}}}_{l}\right)$ (Khalil, 2002). Loosely speaking, being passive means a dynamical system does not produce more energy than it receives, and therefore, it ensures stable behaviors both in free motion and in the case of interactions with passive environments (Schindlbeck and Haddadin, 2015). Although the effect of damping is always dissipating energy from the system, the variation of stiffness will inject extra energy into the system when ${\tilde{q}}_{l}^{\text{T}}{\dot{K}}_{P,l}{\tilde{q}}_{l}$ is positive, and consequently, may break the condition of passivity and drive the system unstable. In the special case of conventional impedance control with fixed gains, i.e. when ${\dot{K}}_{P,l}=0$ , the system under control is essentially stable all the time. Whereas in more general cases, the sign of the term inside the parentheses in Eq. 15 is undefined, and thus the stability is not guaranteed.

Following the analysis above, imposing such a condition

$$\int_{0}^{t}\left(\frac{1}{2}{\tilde{q}}_{l}^{\text{T}}{\dot{K}}_{P,l}{\tilde{q}}_{l}-{\dot{\tilde{q}}}_{l}^{\text{T}}K_{D,l}{\dot{\tilde{q}}}_{l}\right)\text{d}t\leq 0$$ (17)

will make Eq. 16 satisfied again even though the system is under variable stiffness control where ${\dot{K}}_{P,l}\neq 0$ . The inequality Eq. 17 also depicts the principle of the *tank*-based passivity constraint. That is, we may store the dissipated energy into a *virtual* tank and withdraw the energy to cover some non-passive actions without loss of passivity. From the perspective of control theories, the *tank energy* depicts a sort of *passivity margin* which tells the controller how much variation of stiffness can be afforded now.

### 3.2 Stiffness optimization with tank-based constraint

Some observations and considerations on the computation of joint stiffness gains are first provided before presenting our scheme of the stiffness optimization with guaranteed stability.

First of all, the torque command ***τ*** = ***τ***_*ff*_ + ***τ***_*fb*_ is subject to boundary constraints imposed by the actuators, i.e., ***τ*** ∈ [***τ*** ^min^, ***τ*** ^max^]. It is worth noting that the values of the desired contact forces **f** ^*d*^ and the joint stiffness matrix **K**_*P*_ affect each other. This is because the feedforward torques ***τ***_*ff*_, which are mapped from **f** ^*d*^ by Eq. 8, place a constraint on ***τ***_*fb*_ and thus constrain the feasible range of the stiffness gains, i.e., there are

$$\tau^{\min}-\tau_{ff}\leq \underset{\tau_{fb}}{\underset{︸}{K_{P}\tilde{q}+K_{D}\dot{\tilde{q}}}}\leq \tau^{\max}-\tau_{ff}$$

Another observation is that the position-based commands calculated by the whole-body IK controller and the desired contact forces **f** ^*d*^ are actually consistent to achieve the Cartesian reference motions. Therefore, it is the authors’ belief that the computation of the desired contact forces and the joint stiffness is better to be considered together, which should permit more possibilities to achieve desired motions and desired compliance in a coordinated manner.

Additionally, it is worth reminding that the sign of the ${\dot{K}}_{P}$ related term is undefined, dependent on the eigenvalues of ${\dot{K}}_{P}$ . It implies that, from the point of view of passivity, the effect of stiffening some joints may be compensated by relaxing other joints rather than merely draining the energy tanks. Compared with analytical formulations, numerical optimization should be better in exploring the structure of ${\dot{k}}_{P}$ and leads to less conservative results.

Motivated by these thoughts, we propose an optimization over both contact forces and joint stiffness by extending the previous optimization Eq. 6 as follows.

$$\underset{f,{\dot{k}}_{p},\epsilon }{\min}\alpha_{1}\|A_{f}f-b_{f}{\|}^{2}+\alpha_{2}\|f{\|}_{W}^{2}+\alpha_{3}\|k_{P}^{C}\left(k_{p}\right)-k_{P,d}^{C}{\|}_{F}^{2}+\alpha_{4}\|Nk_{p}-k_{p,d}^{\mathrm{track}}{\|}^{2}+\alpha_{5}\|{\dot{k}}_{p}{\|}^{2}+\alpha_{6}\|\epsilon {\|}^{2}$$ (18)

$$\begin{array}{ll} \text{s.t.} & \\ & \;d_{f,\min}\leq C_{f}f\leq d_{f,\max} \end{array}$$ (18a)

$$\;k_{p}\geq k_{p,\min}$$ (18b)

ϵ≥0 (18c)

$$\;|\tau |\leq I\left(\epsilon \right)\tau_{c}$$ (18d)

$$\;\frac{1}{2}{\tilde{q}}_{i}^{\text{T}}{\dot{K}}_{P,i}^{J}{\tilde{q}}_{i}\leq {\dot{\tilde{q}}}_{i}^{\text{T}}K_{D,i}^{J}{\dot{\tilde{q}}}_{i}+\frac{E_{i}^{\mathrm{tank}}}{△T},\;i=\left\{\text{LF, RF, LB, RB}\right\}$$ (18e)

where the first two terms in the cost function Eq. 18 and the first constraint Eq. 18a are exactly from the previous optimization Eq. 6. ${\dot{k}}_{p}\in R^{n}$ is the variation rate of the joint stiffness $k_{p}\in R^{n}$ . At time instant *t*_*k*_, there is approximately

$$k_{p,t_{k}}=k_{p,t_{k-1}}+△T{\dot{k}}_{p,t_{k}}$$

with △*T* being the servo time of the controller, which is typically a small value (in our case it is 5 ms). In this paper, we assume that the stiffness and damping are decoupled among joints. That is, the stiffness matrix $K_{P}^{J}\in R^{n\times n}$ and the damping matrix $K_{D}^{J}\in R^{n\times n}$ are both diagonal matrices with the joint stiffness and the joint damping on their diagonals, respectively. $\epsilon \in R^{n}$ is the slack factors of torque limits that will be introduced later.

The *α*_3_-related term in Eq. 18 aims to track the desired Cartesian stiffness. $K_{P,d}^{C}$ denotes the desired Cartesian stiffness and $K_{P}^{C}\left(k_{p}\right)$ denotes the actual Cartesian stiffness dependent on joint stiffness **k**_*p*_. ‖ ⋅‖_*F*_ denotes the Frobenius norm of matrices. As known in the literature (Albu-Schäffer et al., 2004; Petit and Albu-Schäffer, 2011), there is a relationship between the stiffness matrices in Cartesian space and in joint space as follows

$$K_{P}^{C}\left(k_{p}\right)=J^{+\text{T}}K_{P}^{J}\left(k_{p}\right)J^{+}$$ (19)

where (⋅)^+^ denotes the pseudo-inverse of the Jacobian **J**. The benefit of using Frobenius norm rather than other types of norm is the convenience of transforming the problem of matrix minimization to a standard, convex minimization of a vector (Albu-Schäffer et al., 2004)

$$A_{p}k_{p}=b_{p}$$ (20)

so that the proposed optimization Eq. 18 can be solved as a standard QP problem by many off-the-shelf solvers.

Besides tracking desired Cartesian stiffness, the *α*_4_-related term is to achieve nullspace stiffness where the projector $N\in R^{n\times n}$ maps **k**_*p*_ into the null space of the *α*_3_-related task, that is

$$N=I-A_{p}^{+}A_{p}$$ (21)

where **A**_*p*_ is the same matrix as in Eq. 20. $k_{p,d}^{\mathrm{track}}\in R^{n}$ denotes the desired stiffness in null space, which is calculated from the tracking error of joint positions

$$k_{p,d}^{\mathrm{track}}=\beta {\tilde{q}}^{2}$$ (22)

where $\tilde{q}\in R^{n}$ is the vector of position errors and (⋅)^2^ denotes specially an operation of element-wise square of a vector. The benefit of including this term is, as we have found in many tests, to coordinate the calculated joint stiffness in order to avoid one huge stiffness for some joint while quite small values for the others.

Besides the peak torque limit, there also exists a continuous torque limit ***τ***_*c*_ for many models of actuators (e.g., those used on our robot) that allows for long-term operation without triggering the overheat protection. However, the continuous torque limit is a rather conservative constraint because it is usually less than half of the peak torque limit, and exceeding this limit slightly and occasionally does not prevent continuous operation in practice. Consequently, the inequality Eq. 18, imposes a soft constraint on the reference joint torques by using the relaxed continuous torque limit, where $I\left(\epsilon \right)\in R^{n\times n}$ is a diagonal matrix with the *i*-th element being 1 + *ϵ*_*i*_ (*ϵ*_*i*_ is the *i*-th element of ***ϵ***).

Following the analysis in Section 3.1, we propose to adopt the tank-based passivity constraint to keep the robot stable with stiffness modulation. The tank-based constraints, which are described by the inequalities Eq. 18e, are imposed separately on each leg, where $E_{i}^{\mathrm{tank}}$ denotes the energy stored from the *i*-leg and (⋅)_*i*_ denotes the subvector or the submatrix corresponding to the joints on the *i*-leg. In addition, as pointed out in (Ferraguti et al., 2013), the tank-based constraint is actually not able to preserve the system’s stability in practice unless the tank energy is properly bounded, and therefore, a positive, *application-dependent* upper bound *E*_max_ is required. The selection of a *proper* *E*_max_, however, remains an open problem and it will be selected intuitively later. Eventually, the tank energy at time instant *t*_*k*_ is update by the following rules

$$E_{i,t_{k}}^{\mathrm{tank}}=\left\{\begin{array}{cc} E_{0}\; & \text{if }E_{0}<E_{\max},\\ E_{\max}\; & \text{else} \end{array}\right.$$ (23)

where

$$E_{0}=E_{i,t_{k-1}}^{\mathrm{tank}}+△T\left({\dot{\tilde{q}}}_{i}^{\text{T}}K_{D,i}^{J}{\dot{\tilde{q}}}_{i}-\frac{1}{2}{\tilde{q}}_{i}^{\text{T}}{\dot{K}}_{P,i}^{J}{\tilde{q}}_{i}\right)$$ (24)

Moreover, it is worth remarking that the stability of the variable stiffness locomotion is *always preserved* no matter if the energy tanks are empty or not. When the energy runs out, the optimization Eq. 18 will stop the variation of joint stiffness so that the robot remains passive anyway at the price of losing track of the desired Cartesian stiffness $K_{P,d}^{C}$ .

The *α*_1_, … , *α*_6_ in the optimization Eq. 18 are positive weight factors which should be tuned properly to coordinate all the objectives. The first two terms majorly affect the computation of the desired contact forces, where the *α*_2_-related term regularizes the results. A too strong regularization may cause significant tracking errors and compromise the task performances, and consequently *α*_2_ is usually much smaller than *α*_1_. The computation of the desired joint stiffness is majorly affected by the next three terms. The priority between the *α*_3_-related and the *α*_4_-related terms has been enforced by the nullspace projection and thus these two parameters may be selected similarly in magnitudes, whereas *α*_5_ should be relatively smaller to regularize and smooth the stiffness profiles. *α*_6_ controls the computation of the slack factors ***ϵ*** and, as a result, affects how much the reference joint torques comply with the continuous torque limit. It is usually much larger in the order of magnitudes so that the torque constraints can be obeyed as much as possible. Based on these principles, a few simulation tests should be enough to help decide the proper values of the factors *α*_1_, … , *α*_6_. The selection of parameters used in our tests will be introduced later in Section 4.1.

## 4 Results

To validate the proposed control pipeline, we conduct several experimental tests on our quadruped robot CENTAURO. The results are provided in this section.

### 4.1 Parameters and setup

**FIGURE 4:**
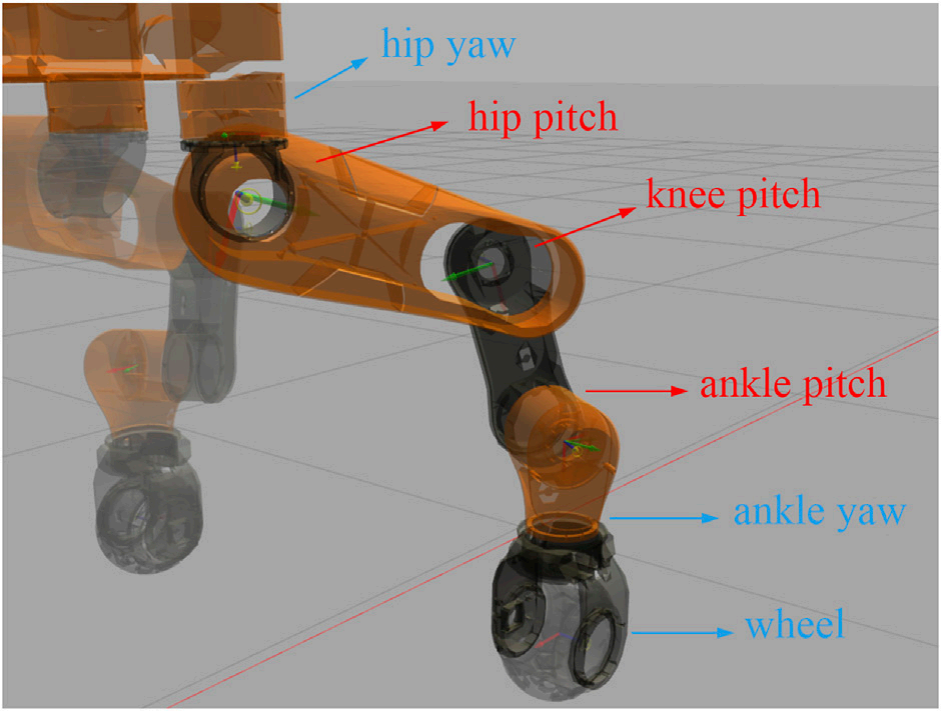
CENTAURO has six joints on each leg. The proposed scheme of stiffness modulation is only applied to the pitch joints on each leg in our experiments, i.e., those marked by red arrows.

CENTAURO is a wheeled-legged quadruped robot designed in our lab (Humanoids and Human Centered Mechatronics, HHCM) at IIT (Kashiri et al., 2019). The robot weights 110 kg and is powered by 42 fully torque-controlled joints where each leg is composed of six actuated joints, namely three pitch joints, two yaw joints and a wheel, as shown in Figure 4. All the modules shown in Figure 2 are implemented as ROS nodes in C++ and run at the indicated rates. The communication between user’s programs and the robot’s hardware is established by *XBotCore*, a real-time (RT) safe robot control framework developed in our lab, which also provides interfaces to non-RT processes (Muratore et al., 2017).

When our robot walks on unknown terrains using touchdown adaptations, the huge impulse with the ground majorly appears on the *z*-axis of the world frame, the magnitude of which may exceed 600 N sometimes. Aiming to mainly reduce the contact impulse in locomotion, we apply the proposed optimal stiffness modulation only to the 12 pitch joints of the legs (marked by red arrows in Figure 4). Those joints contribute the most to the Cartesian stiffness on the *z*-axis and, by modulating their stiffness online, the balance between tracking performance and desired compliance at the moments of contact should be achieved. Correspondingly, the desired Cartesian stiffness matrix in the optimization Eq. 18 becomes $K_{P,d}^{C}\in R^{4\times 4}$ , only containing the stiffness on the *z*-axis of the world frame for the four legs. Additionally, the decision variables in the optimization Eq. 18 become $f\in R^{12}$ , ${\dot{K}}_{p}\in R^{12}$ and $\epsilon \in R^{12}$ , which also results in a smaller problem to solve.

For selection of parameters, the torque limit $\tau_{c}\in R^{12}$ , as reported in (Kashiri et al., 2019), is set to be $\tau_{c}={\left[\tau_{c,1},\ldots ,\tau_{c,4}\right]}^{\text{T}}$ where ***τ***_*c*,*i*_ = [123, 123, 46] Nm. Since we mainly consider the effects of modulating joint stiffness towards locomotion at present, the damping matrix is temporarily left to be constant in the following tests, being $K_{D}^{J}=\text{diag}\left(K_{D,1},\ldots ,K_{D,4}\right)$ where **K**_*D*,*i*_ = diag ([40, 40, 20]) Nms/rad. As mentioned in Section 3.2, there is no systematic way to select the upper bound of the tank energy *E*_max_. It is set to be 5 J in our cases based on the observation that 5 J is a relatively conservative bound but allows to keep the tank energy positive, i.e., enabling continuous modulation of the joint stiffness gains, in most experimental trials. The design of the desired Cartesian stiffness will be introduced later in Section 4.2.1.

Unless otherwise stated, other parameters used in the following experiments are listed in Table 2.

**TABLE 2:** Parameter list.

Parameter	Value	Parameter	Value
*α*_1_	1	*β*	1 × 10^5^
*α*_2_	1 × 10^–4^	**k**_*p*, min_	100**I**_12_ Nm/rad
*α*_3_	1	*f*_*up*_	50 N
*α*_4_	1	△*T*	0.005 s
*α*_5_	1 × 10^–1^	*E*_max_	5 J
*α*_6_	1 × 10^6^		

### 4.2 Experimental results

**FIGURE 5:**
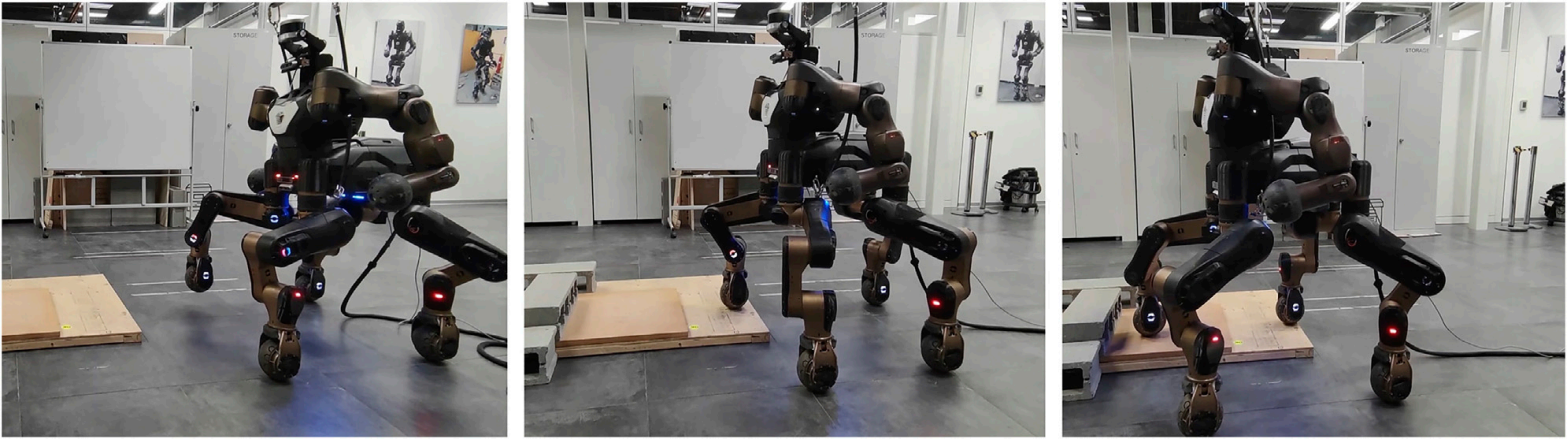
Snapshots of the variable stiffness locomotion, where the robot needs to walk up two steps in the workspace without knowing the terrain geometries beforehand.

The experimental results of applying the proposed control pipeline on our robot CENTAURO are displayed in this part. The workspace consists of two wooden steps as shown in Figure 5, where the bottom step is about 6 cm high and the upper step is about 4 cm high. The robot needs to walk up the two steps without knowing the height maps beforehand. Although the task is not quite challenging, it provides variations of several centimeters in the height maps, which enables to demonstrate the improvements of the variable stiffness locomotion and the effectiveness of the developed contact-triggered touchdown adaptation. Please refer to the supplementary video for the whole execution of the following trials.

#### 4.2.1 Variable stiffness locomotion

The results of using the proposed control pipeline are displayed in this section.

**FIGURE 6:**
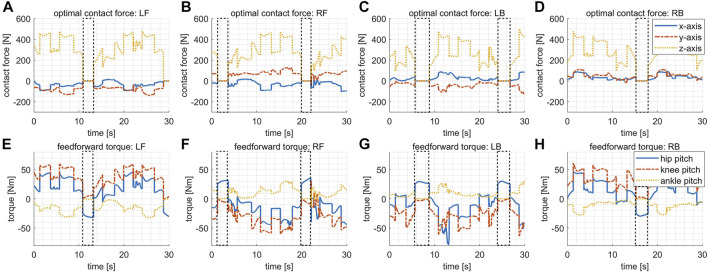
The desired contact forces and the feedforward torques in the variable stiffness locomotion. The dashed boxes indicate the periods when the corresponding leg is in swing. **(A)** The desired contact force for the leg LF. **(B)** The desired contact force for the leg RF. **(C)** The desired contact force for the leg LB. **(D)** The desired contact force for the leg RB. **(E)** The feedforward torques for the leg LF. **(F)** The feedforward torques for the leg RF. **(G)** The feedforward torques for the leg LB. **(H)** The feedforward torques for the leg RB.

Figure 6 shows the optimal contact forces **f** ^*d*^ and the feedforward reference torques ***τ***_*ff*_. The dashed boxes indicate the periods when the corresponding leg is in swing, within which the expected contact forces should be 0. However, the corresponding feedforward reference torques are not 0 within the swing phases since the compensation for gravity and Coriolis effects has already been considered by the mapping Eq. 8. It is worth noting that the swing periods are not a constant value because the robot adapts to early or late touchdown rather than relying on nominal transitions between gait phases. The effectiveness of the touchdown adaptation will be better illustrated later by the last trial, where we disable this function and let the robot perform the same task.

**FIGURE 7:**
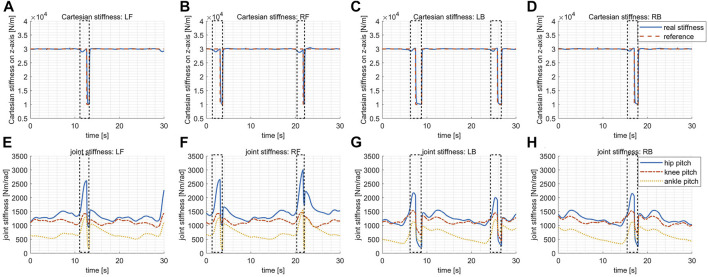
The Cartesian stiffness and the corresponding optimal joint stiffness in the variable stiffness locomotion. The dashed boxes indicate the periods when the corresponding leg is in swing. **(A)** The desired/actual Cartesian stiffness for the leg LF. **(B)** The desired/actual Cartesian stiffness for the leg RF. **(C)** The desired/actual Cartesian stiffness for the leg LB. **(D)** The desired/actual Cartesian stiffness for the leg RB. **(E)** The optimal joint stiffness for the leg LF. **(F)** The optimal joint stiffness for the leg RF. **(G)** The optimal joint stiffness for the leg LB. **(H)** The optimal joint stiffness for the leg RB.

The design of the desired Cartesian stiffness introduced in Eq. 18 depends on specific applications, expressing the expected compliance of the robot when interactions happen. For instance, the stiffness gains may be adapted online based on the detected environments and expected interactions when exteroceptive sensors are available. In our experiments without perception aided, the desired Cartesian stiffness is designed in a pattern simply dependent on gait phases as shown by the dashed red lines in Figure 7A–D. To be specific, the desired stiffness gain reduces to a smaller value (1 × 10^4^ N/m) at the second half of swing phases while remaining at a higher value (3 × 10^4^ N/m) at the other times, which aims to balance the requirements of supporting the body steadily and touching down compliantly.

The optimized joint stiffness gains for each leg are shown in Figure 7E–H, respectively. The stiffness gain of the ankle pitch joint is in general much smaller than the gains of the hip pitch and the knee pitch joints because of the smaller torque capacity of the ankle pitch joint, as explained previously in Section 4.1. The joint stiffness gains increase at the first half of the swing phase majorly due to the variation of the leg configuration, and thus the variation of the Jacobian matrix involved in Eq. 19, which requires higher gains to maintain the same level of Cartesian stiffness. Whereas at the second half of the swing phase, the joint stiffness gains dropped rapidly aiming to track the lower reference stiffness in Cartesian space. By checking the plots Figure 7A–D, it is clear that the actual Cartesian stiffness have managed to track the desired values with reasonably good precision.

**FIGURE 8:**
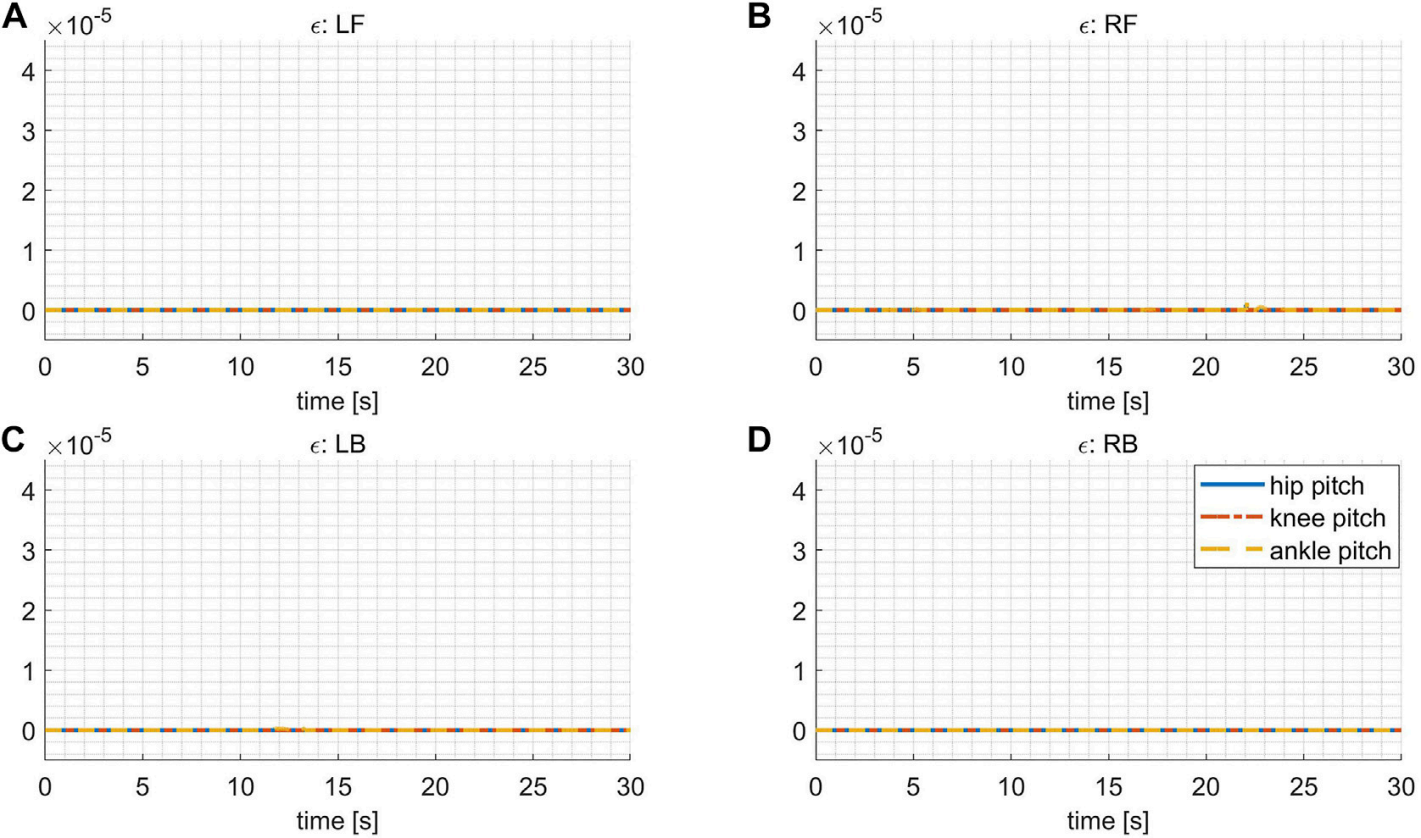
The slack factor ***ϵ*** of continuous torque limits in the variable stiffness locomotion. **(A)** ***ϵ*** for the leg LF. **(B)** ***ϵ*** for the leg RF. **(C)** ***ϵ*** for the leg LB. **(D)** ***ϵ*** for the leg RB.

The slack factor ***ϵ*** in Eq. 18d controls how much the joint torques comply with the continuous torque limit. The closer of ***ϵ*** to 0, the better the reference joint torques comply with the constraints. As explained in Section 3.2, the weight factor *α*_6_, which controls the computation of ***ϵ***, is selected to be a relatively huge value in order to obey the torque constraints as much as possible. The values of ***ϵ*** shown in Figure 8 are all sufficiently close to 0, coinciding with our expectation, which implies that all the actuators work within the continuous torque range with only negligible violations.

**FIGURE 9:**
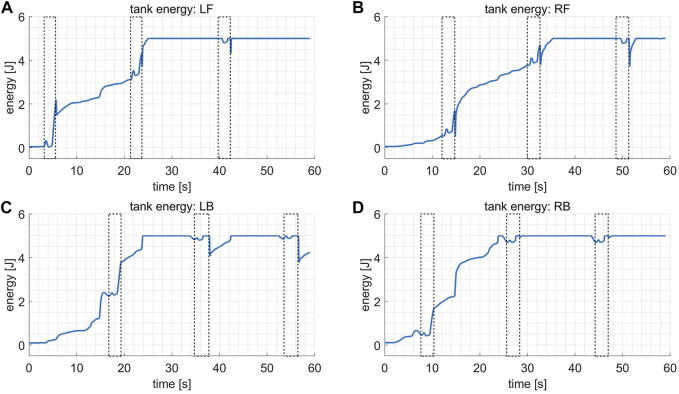
The plots of the tank energy in the variable stiffness locomotion, where the dashed boxes indicate the swing phases. **(A)** Tank energy for the leg LF. **(B)** Tank energy for the leg RF. **(C)** Tank energy for the leg LB. **(D)** Tank energy for the leg RB.

Figure 9 shows how the energy stored in virtual tanks, i.e. the passivity margin, varies during the test, where the dashed boxes indicate the swing phases. It is observed that a sudden drop in the tank energy usually occurs at the end of the swing phase due to the rapid rise of the joint stiffness gains, as expressed in Figure 7, which consumes the stored passivity margin. However, in general, the energy remains positive and increases until it reaches the upper bound (5 J), implying that it is always affordable for some non-passive actions when modulating the joint stiffness without undermining the robot’s stability.

#### 4.2.2 Comparative tests

We also compare the proposed control pipeline with other strategies on the robot and the results will be provided in the following part.

##### 4.2.2.1 High stiffness locomotion

Compared with locomotion with fixed joint stiffness, the proposed variable stiffness locomotion may achieve a better balance between tracking performance and contact compliance. To this end, we repeat the task above with different configurations of joint stiffness.

**FIGURE 10:**
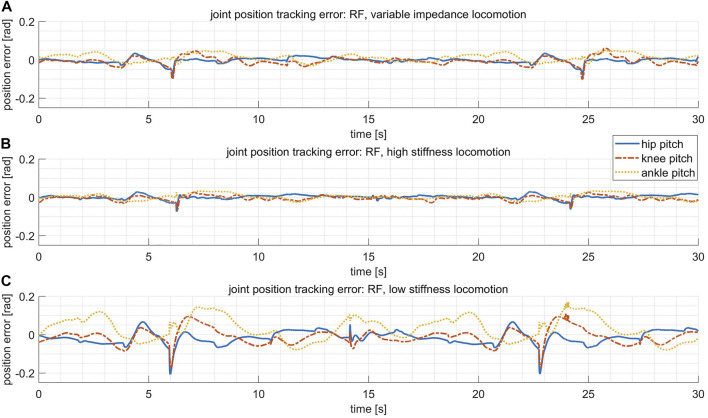
The joint position tracking error of the leg RF in three comparative tests. **(A)** In the variable stiffness locomotion. **(B)** In the high stiffness locomotion. **(C)** In the low stiffness locomotion.

**FIGURE 11:**
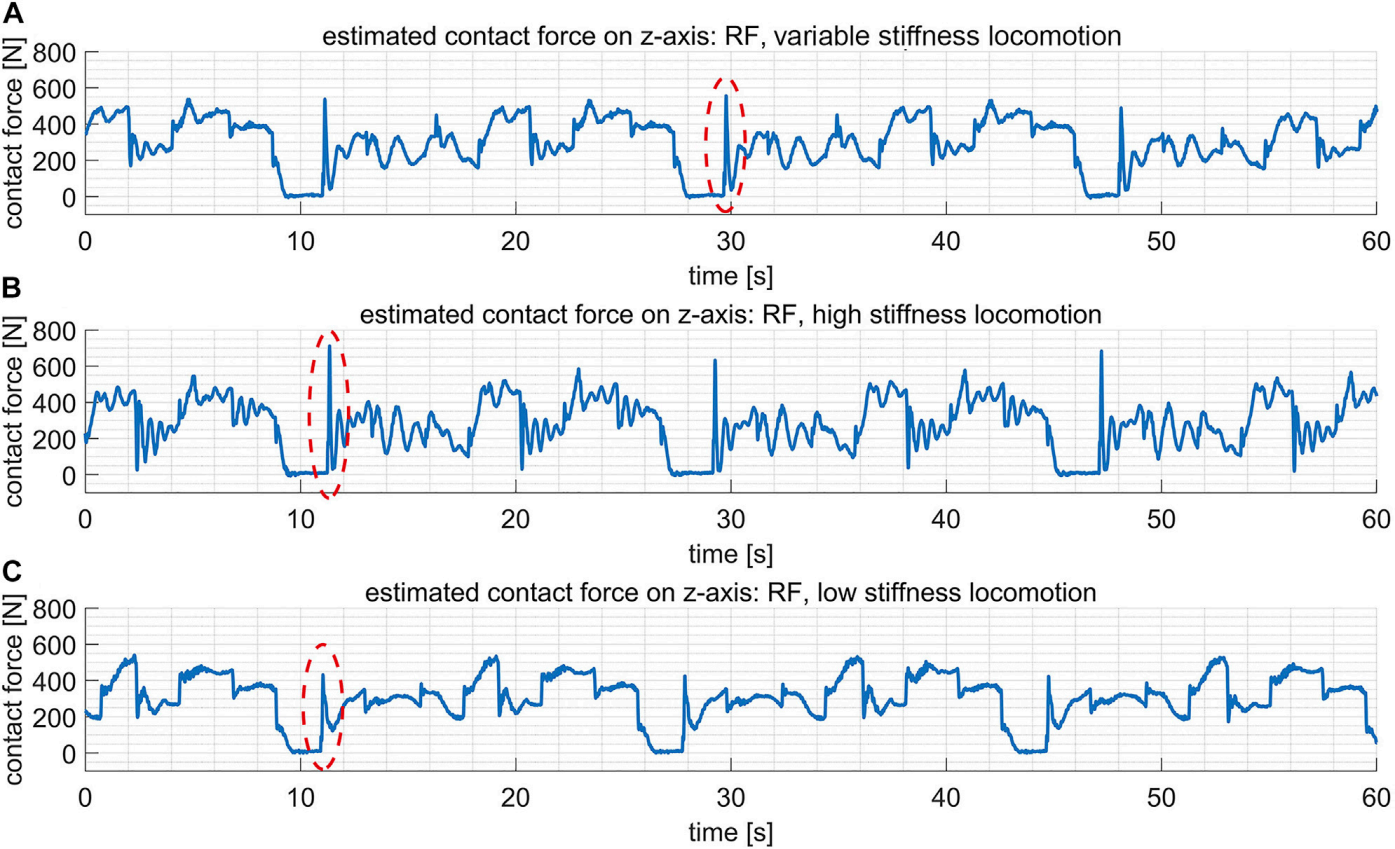
The estimated contact force (on *z*-axis of the world frame) of the leg RF in three comparative tests. The maximum contact forces at the moment of contact are marked by the red ellipses. **(A)** In the variable stiffness locomotion. **(B)** In the high stiffness locomotion. **(C)** In the low stiffness locomotion.

In this test, the stiffness for the hip pitch, knee pitch and ankle pitch joints on each leg are fixed to be [2000, 2000, 1,000] Nm/rad. Figure 10 reveals that the tracking errors of joint positions are roughly on the same scale for the variable stiffness locomotion and the high stiffness locomotion. However, by checking Figure 11, it shows that the variable stiffness locomotion has smaller impulse when the swing leg touches down, which can also be observed intuitively in the supplementary video. The maximum force at the moment of contact (marked by red ellipses) has reduced from 711 N in high stiffness locomotion to 556 N in variable stiffness locomotion.

##### 4.2.2.2 Low stiffness locomotion

In this test, the stiffness for the hip pitch, knee pitch and ankle pitch joints on each leg are fixed to be [600, 600, 300] Nm/rad. The impulse with the ground has even reduced in this case as shown in Figure 11, however, the tracking errors of joint positions become much larger in Figure 10. Actually, the robot failed to walk up the step because of the terrible tracking performance, as revealed in the supplementary video.

##### 4.2.2.3 Locomotion without touchdown adaptation

In order to validate the effectiveness of the implemented touchdown adaptation, we disable this function and then repeat the variable stiffness locomotion in the same task. To be specific, the Cartesian motion planner will ignore any *contact signal* and transit from the Swing Phase *directly* to the All-Stance Phase after a period of *t*_*s*_ (seen in Figure 3). The execution of this test is included in the supplementary video, which reveals that the robot failed to accomplish the task without the touchdown adaptation.

## 5 Discussion and future work

As mentioned in Section 1, optimal modulation of joint stiffness for quadruped robots is studied in (Heijmink et al., 2017), where the desired stiffness profiles are trained by learning algorithms. The offline training in simulations allows optimizing the energy efficiency when computing for the desired stiffness profiles, whereas our scheme presented in this paper cannot account for the overall energy consumption. The scheme proposed in (Heijmink et al., 2017), however, is only tested in simulations without hardware-based demonstrations, perhaps due to the considerable time consumption of the training process and the difficulties in sim-to-real practice. Another deficiency is that there is no theoretical consideration of the stability issues under variable stiffness control, which implies that the learned modulation may lead the robot to go divergence in some corner cases.

A variable stiffness controller with guaranteed stability is developed in (Angelini et al., 2019) for the quadruped robot ANYmal. In order to prove the stability, it requires decomposing the dynamics of the base into decoupled, one-DoF sub-systems in different directions, which may not be valid for complex robots with an upper body and dual arms such as CENTAURO. In contrast, an energy tank-based constraint is employed in this work to prove the stability of robots with time-varying stiffness gains, which requires fewer assumptions and approximations to validate the proof.

The tank-based approach is introduced by (Ferraguti et al., 2013) to study the issue of stability for manipulators with time-varying joint stiffness. However, one drawback of the proposed controller there is that the stiffness will fall back to a predefined constant value whenever the tank is empty. Such a sudden change corresponds to an infinitely large variation rate of the stiffness and may cause oscillations of the motors, which should be avoided in real applications. In contrast, our scheme simply stops the variation of joint stiffness whenever the tank is empty so there are no discontinuities in the generated stiffness profiles.

In this paper, we assume the stiffness and damping matrices, i.e. $K_{P}^{J}$ and $K_{D}^{J}$ , to be diagonal, which may prohibit tracking the desired Cartesian stiffness precisely at a given joint configuration, as pointed out in (Albu-Schäffer et al., 2004). A possible solution is proposed in (Petit and Albu-Schäffer, 2011) to optimize the coupled stiffness matrix directly by the *matrix nearness* technique. However, that matrix-optimization approach seems impossible to account for constraints such as torque limits and the energy tank-based passivity constraint, which may produce infeasible or unstable behaviors for the real robots. An alternative method to mitigate the aforementioned restriction is proposed in (Albu-Schäffer et al., 2004), where the arm configurations are optimized together with the joint stiffness. Since the stability issue is not considered in (Albu-Schäffer et al., 2004), it is an interesting future direction to extend the developed scheme in this work by including an optimization of leg configurations, which should better achieve the desired Cartesian stiffness with guaranteed stability.

We present a control pipeline in this paper that enables our quadruped robot to walk on moderately uneven terrains robustly and compliantly without perception aided. One of our ongoing topics is to combine the proposed locomotion controller with perception in order to traverse more challenging environments. Due to some properties of the objects, the light conditions or the noise from the sensors, the perception may introduce uncertainties of about several centimeters in the terrain information, which, as demonstrated in the experiments, can be well solved by the proposed control pipeline. That work will eventually allow our quadruped robot CENTAURO overcoming more challenging terrains with various obstacles.

## Data Availability

The original contributions presented in the study are included in the article/Supplementary Material, further inquiries can be directed to the corresponding author.

## References

[B1] Albu-Schäffer A. Fischer M. Schreiber G. Schoeppe F. Hirzinger G. (2004). “Soft robotics: What cartesian stiffness can we obtain with passively compliant, uncoupled joints?,” in International Conference on Intelligent Robots and Systems (IROS), 3295–3301. Google Scholar

[B2] Angelini F. Xin G. Wolfslag W. J. Tiseo C. Mistry M. Garabini M. (2019). “Online optimal impedance planning for legged robots,” in International Conference on Intelligent Robots and Systems (IROS), 6028–6035. 10.1109/iros40897.2019.8967696 | Google Scholar

[B3] Bellicoso C. D. Bjelonic M. Wellhausen L. Holtmann K. Günther F. Tranzatto M. (2018). Advances in real-world applications for legged robots. J. Field Robot. 35, 1311–1326. 10.1002/rob.21839 10.1002/rob.21839 | Google Scholar

[B4] Bledt G. Powell M. J. Katz B. Di Carlo J. Wensing P. M. Kim S. (2018a). “Mit cheetah 3: Design and control of a robust, dynamic quadruped robot,” in International Conference on Intelligent Robots and Systems (IROS), 2245–2252. 10.1109/iros.2018.8593885 | Google Scholar

[B5] Bledt G. Wensing P. M. Ingersoll S. Kim S. (2018b). “Contact model fusion for event-based locomotion in unstructured terrains,” in International Conference on Robotics and Automation (ICRA), 4399–4406. 10.1109/icra.2018.8460904 | Google Scholar

[B6] Camurri M. Fallon M. Bazeille S. Radulescu A. Barasuol V. Caldwell D. G. (2017). Probabilistic contact estimation and impact detection for state estimation of quadruped robots. IEEE Robot. Autom. Lett. 2, 1023–1030. 10.1109/lra.2017.2652491 10.1109/lra.2017.2652491 | Google Scholar

[B7] Ferraguti F. Secchi C. Fantuzzi C. (2013). “A tank-based approach to impedance control with variable stiffness,” in International Conference on Robotics and Automation (ICRA), 4948–4953. 10.1109/icra.2013.6631284 | Google Scholar

[B8] Focchi M. Del Prete A. Havoutis I. Featherstone R. Caldwell D. G. Semini C. (2017). High-slope terrain locomotion for torque-controlled quadruped robots. Auton. Robots 41, 259–272. 10.1007/s10514-016-9573-1 10.1007/s10514-016-9573-1 | Google Scholar

[B9] Gehring C. Coros S. Hutter M. Bloesch M. Hoepflinger M. A. Siegwart R. (2013). “Control of dynamic gaits for a quadrupedal robot,” in International Conference on Robotics and Automation (ICRA), 3287–3292. 10.1109/icra.2013.6631035 | Google Scholar

[B10] Heijmink E. Radulescu A. Ponton B. Barasuol V. Caldwell D. G. Semini C. (2017). “Learning optimal gait parameters and impedance profiles for legged locomotion,” in International Conference on Humanoid Robots (Humanoids), 339–346. 10.1109/humanoids.2017.8246895 | Google Scholar

[B11] Hogan N. (1985). Impedance control: An approach to manipulation: Part III—Applications. J. Dyn. Syst. Meas. Control 107, 17–24. 10.1115/1.3140701 10.1115/1.3140701 | Google Scholar

[B12] Kashiri N. Baccelliere L. Muratore L. Laurenzi A. Ren Z. Hoffman E. M. (2019). Centauro: A hybrid locomotion and high power resilient manipulation platform. IEEE Robot. Autom. Lett. 4, 1595–1602. 10.1109/lra.2019.2896758 10.1109/lra.2019.2896758 | Google Scholar

[B13] Khalil H. K. (2002). Nonlinear systems. Third Edition. Upper Saddle River, New Jersey, USA: Prentice-Hall. Google Scholar

[B14] Klamt T. Rodriguez D. Schwarz M. Lenz C. Pavlichenko D. Droeschel D. (2018). “Supervised autonomous locomotion and manipulation for disaster response with a centaur-like robot,” in International Conference on Intelligent Robots and Systems (IROS), 1–8. 10.1109/iros.2018.8594509 | Google Scholar

[B15] Kronander K. Billard A. (2016). Stability considerations for variable impedance control. IEEE Trans. Robot. 32, 1298–1305. 10.1109/tro.2016.2593492 10.1109/tro.2016.2593492 | Google Scholar

[B16] Laurenzi A. Hoffman E. M. Muratore L. Tsagarakis N. G. (2019). “Cartesi/o: A ros based real-time capable cartesian control framework,” in International Conference on Robotics and Automation (ICRA), 591–596. 10.1109/icra.2019.8794464 | Google Scholar

[B17] Lee H. Hogan N. (2014). Time-varying ankle mechanical impedance during human locomotion. IEEE Trans. Neural Syst. Rehabil. Eng. 23, 755–764. 10.1109/tnsre.2014.2346927 PubMed Abstract | 10.1109/tnsre.2014.2346927 | Google Scholar 25137730

[B18] Lee J. Hwangbo J. Wellhausen L. Koltun V. Hutter M. (2020). Learning quadrupedal locomotion over challenging terrain. Sci. Robot. 5, eabc5986. 10.1126/scirobotics.abc5986 PubMed Abstract | 10.1126/scirobotics.abc5986 | Google Scholar 33087482

[B19] Mastalli C. Focchi M. Havoutis I. Radulescu A. Calinon S. Buchli J. (2017). “Trajectory and foothold optimization using low-dimensional models for rough terrain locomotion,” in International Conference on Robotics and Automation (ICRA), 1096–1103. 10.1109/icra.2017.7989131 | Google Scholar

[B20] Mistry M. Buchli J. Schaal S. (2010). “Inverse dynamics control of floating base systems using orthogonal decomposition,” in International Conference on Robotics and Automation (ICRA), 3406–3412. 10.1109/robot.2010.5509646 | Google Scholar

[B21] Muratore L. Laurenzi A. Hoffman E. M. Rocchi A. Caldwell D. G. Tsagarakis N. G. (2017). “Xbotcore: A real-time cross-robot software platform,” in International Conference on Robotic Computing, 77–80. 10.1109/irc.2017.45 | Google Scholar

[B22] Orin D. E. Goswami A. Lee S.-H. (2013). Centroidal dynamics of a humanoid robot. Auton. Robots 35, 161–176. 10.1007/s10514-013-9341-4 10.1007/s10514-013-9341-4 | Google Scholar

[B23] Ott C. Roa M. A. Hirzinger G. (2011). “Posture and balance control for biped robots based on contact force optimization,” in International Conference on Humanoid Robots (Humanoids), 26–33. 10.1109/humanoids.2011.6100882 | Google Scholar

[B24] Petit F. Albu-Schäffer A. (2011). “Cartesian impedance control for a variable stiffness robot arm,” in International Conference on Intelligent Robots and Systems (IROS), 4180–4186. 10.1109/iros.2011.6094736 | Google Scholar

[B25] Righetti L. Buchli J. Mistry M. Kalakrishnan M. Schaal S. (2013). Optimal distribution of contact forces with inverse-dynamics control. Int. J. Robotics Res. 32, 280–298. 10.1177/0278364912469821 10.1177/0278364912469821 | Google Scholar

[B26] Schindlbeck C. Haddadin S. (2015). “Unified passivity-based cartesian force/impedance control for rigid and flexible joint robots via task-energy tanks,” in International Conference on Robotics and Automation (ICRA), 440–447. 10.1109/icra.2015.7139036 | Google Scholar

[B27] Sentis L. Park J. Khatib O. (2010). Compliant control of multicontact and center-of-mass behaviors in humanoid robots. IEEE Trans. Robot. 26, 483–501. 10.1109/tro.2010.2043757 10.1109/tro.2010.2043757 | Google Scholar

[B28] Siciliano B. Sciavicco L. Villani L. Oriolo G. (2009). Modelling, planning and control. Advanced textbooks in control and signal processing. Springer. Google Scholar

[B29] Stephens B. J. Atkeson C. G. (2010). “Dynamic balance force control for compliant humanoid robots,” in International Conference on Intelligent Robots and Systems (IROS), 1248–1255. 10.1109/iros.2010.5648837 | Google Scholar

[B30] Winkler A. W. Farshidian F. Pardo D. Neunert M. Buchli J. (2017). Fast trajectory optimization for legged robots using vertex-based zmp constraints. IEEE Robot. Autom. Lett. 2, 2201–2208. 10.1109/lra.2017.2723931 10.1109/lra.2017.2723931 | Google Scholar

[B31] Wu Y. Zhao F. Kim W. Ajoudani A. (2020a). An intuitive formulation of the human arm active endpoint stiffness. Sensors 20, 5357. 10.3390/s20185357 10.3390/s20185357 | Google Scholar PMC7570772 32962084

[B32] Wu Y. Zhao F. Tao T. Ajoudani A. (2020b). A framework for autonomous impedance regulation of robots based on imitation learning and optimal control. IEEE Robot. Autom. Lett. 6, 127–134. 10.1109/lra.2020.3033260 10.1109/lra.2020.3033260 | Google Scholar

[B33] Zhao X. You Y. Laurenzi A. Kashiri N. Tsagarakis N. (2021). “Locomotion adaptation in heavy payload transportation tasks with the quadruped robot centauro,” in International Conference on Robotics and Automation (ICRA), 5028–5034. 10.1109/icra48506.2021.9561720 | Google Scholar

[B34] Zollo L. Siciliano B. Laschi C. Teti G. Dario P. (2003). An experimental study on compliance control for a redundant personal robot arm. Robotics Aut. Syst. 44, 101–129. 10.1016/s0921-8890(03)00042-3 10.1016/s0921-8890(03)00042-3 | Google Scholar

